# A Panel of Eight miRNAs Is Deregulated in HTLV-2 Infected PBMCs and BJABGu Cell Line

**DOI:** 10.3390/ijms23147583

**Published:** 2022-07-08

**Authors:** Elisabetta Pilotti, Attilio Cannata, Giacomo Magnani, Fabio Bignami, Andrea Corsi, Maria Teresa Valenti, Mariam Shallak, Greta Forlani, Maria Grazia Romanelli

**Affiliations:** 1 Department of Neurosciences, Biomedicine and Movement Sciences, University of Verona, 37124 Verona, Italy; elisabetta.pilotti@univr.it (E.P.); andrea.corsi@univr.it (A.C.); mariateresa.valenti@univr.it (M.T.V.); 2 L.C. Laboratori Campisi s.r.l., 96012 Avola, Italy; attilio.cannata@gmail.com; 3 Unit of Infectious Diseases, Azienda USL-IRCCS, 42121 Reggio Emilia, Italy; giacomo.magnani51@gmail.com; 4 Department of Clinical Sciences, University of Milano, 20121 Milano, Italy; fabio.bignami@gmail.com; 5 Laboratory of General Pathology and Immunology “Giovanna Tosi”, Department of Medicine and Surgery, University of Insubria, 21100 Varese, Italy; mshallak@uninsubria.it (M.S.); greta.forlani@uninsubria.it (G.F.)

**Keywords:** HTLV-2, miRNA, Tax, RUNX, host-virus interactions, cell signaling pathways

## Abstract

Despite human T-cell leukemia virus type 1 (HTLV-1) and HTLV-2 being retroviruses closely related at a genomic level, HTLV-2 differs from HTLV-1 in terms of pathogenicity in both single infection and coinfection contexts. Moreover, the HTLV-2 association with clinical outcomes is still debated and several mechanisms underlying HTLV-2 infection remain unexplored as well. Cellular miRNAs are key factors in the post-transcriptional regulation of gene expression and they are known to be potential targets for several pathogens to control the host microenvironment and, in particular, escape immune responses. Here, we identified a HTLV-2-related signature of eight miRNAs (miR-125a-3p, miR-381-3p, miR-502-5p, miR-708-5p, miR-548d-5p, miR-548c-5p, miR-1-3p, and miR-511-5p) in both HTLV-2 infected PBMC and BJABGu cell lines. Altered miRNA expression patterns were correlated with the impairment of Th cell differentiation and signaling pathways driven by cytokines and transcriptional factors such as the Runt-related transcription factor (RUNX) family members. Specifically, we demonstrated that the RUNX2 protein was significantly more expressed in the presence of Tax-2 compared with Tax-1 in an in vitro cell model. To the best of our knowledge, these data represent the first contribution to elucidating the HTLV-2 mediated alteration of host cell miRNA profiles that may impact on HTLV-2 replication and persistent infection.

## 1. Introduction

MiRNAs are non-coding regulatory RNAs that act at a post-transcriptional level in various cellular processes (i.e., cellular development, differentiation, and death) by inhibiting the gene expression via targeting the specific sequences of mRNAs [1]. Given these properties, the host cell miRNA expression is profoundly altered by viruses to favor their own survival [2]. Furthermore, miRNAs have been reported to contribute to the carcinogenesis triggered by oncoviruses, including human retroviruses [3]. At first, miRNAs emerged as cellular factors involved in the defense against retroviral infections, but they have also been proven to be targeted by viruses in order to overwhelm immune responses [4,5,6]. Specifically, HIV-1 and the human T-cell leukemia virus type 1 (HTLV-1) were found to engage host miRNAs to modify the behavior of infected cells and neighboring uninfected cells in order to promote viral replication and counteract immune responses [7,8].

Among the four types of HTLVs identified so far, HTLV-1 and type 2 (HTLV-2) are the most prevalent ones worldwide [9,10]. HTLV infection is lifelong and is primarily transmissible through breastfeeding, sexual intercourse, injection drug use, and blood transfusion [11]. Even though HTLV-2 is closely related to type 1 through a similar genomic organization, including long terminal repeat (LTR); *gag, pol,* and *env* genes; and regulatory and accessory elements [12], they significantly diverge in clinical manifestations. HTLV-1 causes severe adult T-cell leukemia/lymphoma (ATLL) and tropical spastic paraparesis/HTLV-1 associated myelopathy (TSP/HAM) in up to 6–7% of infected individuals [13], whereas HTLV-2 has not yet been linked to T-cell malignancy [12,14]. HTLV-2 infection has been associated with peripheral neuropathy, but the debate is still open because of contrasting data in the literature [15,16]. However, it has been generally accepted that HTLV-2 could negatively interfere with HIV-1 replication, leading to a delayed progression toward AIDS [17,18]. As the alteration of miRNAs expression triggered by HTLV-2 infection might deeply affect its interaction with HIV-1, investigating the miRNA signature in the contest of HTLV-2 infection may open a new perspective in the study of HIV-1 disease progression.

Both HTLV-1 and HTLV-2 survive through a persistent clonal expansion of infected cells; however, unlike HTLV-1, HTLV-2 exhibits a predominant transformation of the CD8+ T cell subset, which harbors the majority of HTLV-2 proviral load [19]. Cellular immortalization in vivo, as well as viral pathogenesis, are primarily mediated by Tax-1 and Tax-2 transactivating proteins, whose effects on the cellular pathways are divergent [20,21,22,23,24]. Although Tax-1 and Tax-2 show an amino acid similarity of 85%, Tax-2 differs in recruiting host co-activators to enhance LTR transcription and signal transduction compared with Tax-1 [12]. Several cellular pathways are deregulated by Tax proteins, involving AP1, CREB/ATF, Serum response factor (SRF), and NF-kB factors [25]. By investigating the Tax mediated NF-kB activation, we and others identified a number of host factors recruited in complexes with the viral proteins highlighting the differences between the Tax-1 and Tax-2 host factor interactions [26,27,28,29,30]. In addition, Tax-2 shows a primarily cytoplasmic distribution, while Tax-1 is abundant in the nucleus [31,32,33]. Among the four serotypes of HTLV-2 (a-d) that have been identified so far, Tax-2b is the closest to Tax-1 [34].

While MiRNA profiling has been extensively investigated in the context of HTLV-1 infection [5,28,35,36,37,38,39], evidence of HTLV-2’s impact on the miRNA expression is almost absent, except for only one study, which reported a profile of eight miRNAs common to both CD4+ T cells from HTLV-2 and HIV-1 mono-infected patients [40]. In the case of HTLV-1, alteration of the miRNA expression has been shown to be associated with the development of ATL and HAM/TSP disorders [5].

Here, we identified a unique signature of miRNAs related to HTLV-2 infection that might correlate with known molecular pathways critical for the persistence of HTLV-2 replication and the interactions with coinfected pathogens such as HIV-1.

## 2. Results

### 2.1. Identification of Distinct miRNAs Expression Profiles in PBMCs from Mono-HTLV-2 Positive Subjects and in BJABGu Cells

The expression levels of 377 miRNAs were determined in a pool of PBMCs from six HTLV-2-mono-infected subjects and in the BJABGu cell line, both harboring HTLV strains belonging to the subtype 2b. The MiRNA expression analysis was conducted using real-time quantitative PCR and the 2^−ΔΔCt^ method to calculate the relative changes in expression between the infected and uninfected samples.

**Figure 1:**
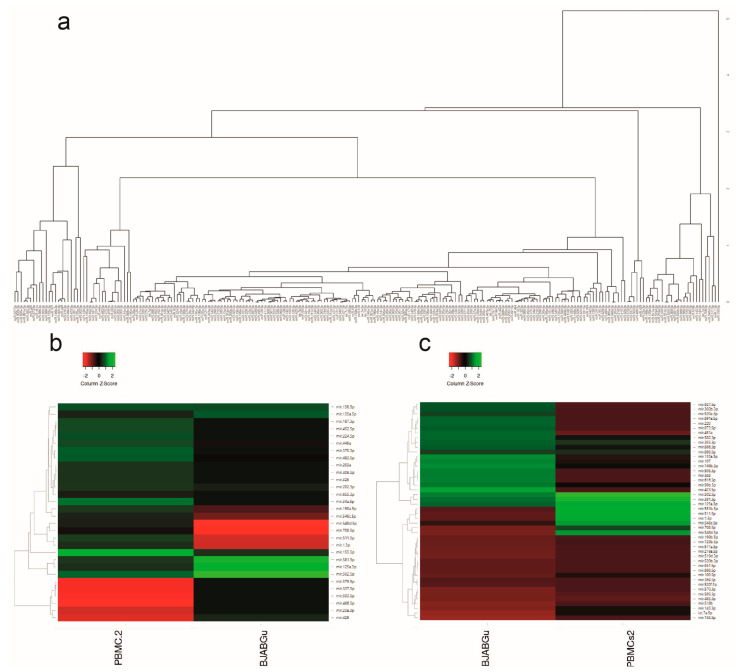
Dendrogram and heat maps of the differentially expressed miRNAs. (**a**) The hierarchical clustering dendrogram shows the similarities between the expression profiles of the 226 dysregulated miRNAs. (**b**) The heat map of miRNAs whose expression levels varied by at least 1 log10 (fold change (FC)) in HTLV-2-infected PBMCs (first column) and profile of corresponding miRNAs altered in BJABGu (second column). (**c**) The heat map of miRNAs with [log10 (FC) > |1|] in BJABGu (first column) and the profile with miRNAs modulated in the HTLV-2-infected PBMCs (second column). Each colored block represents the expression of 1 miRNA (labeled on the right) in the indicated sample. PCR expression signals are converted into color (green, high signal; red, low signal). Color intensities are proportional to the variation of expression, as indicated in the scale bar: values ranged from −2 to + 2 (z score).

We found 226 dysregulated miRNAs in at least one of two infected samples (Log_10_ not equal to 0.00) (Figure 1). Hierarchical clustering analyses evidenced pairs of miRNAs that showed striking similarities in the altered expression (i.e., miR-409-5p/miR-203, miR-203/miR-325, miR-579/miR-29c, miR-576-3p/miR-146b-5p, miR-484/miR-16, miR-584-3p/miR-204, and miR-379/miR-337-5p), whereas miRNAs (i.e., miR-520e, miR-886-5p, miR-502-5p, miR-34a, miR-100, miR-143, and miR-155) differed significantly within each infected sample (Figure 1a).

By applying a fold change (FC) threshold greater than 1 log_10_ in both directions (up and down) 30 and 46 miRNAs in HTLV-2-infected PBMCs and BJABGu cells, respectively, were found. Figure 1 displays the heat map of 30 miRNAs whose expression levels varied by at least 1 log10 (FC) in HTLV-2-infected PBMCs compared with BJABGu (Figure 1b) and the heat map of 46 miRNA with at least 1 log10 (FC) in BJABGu compared with HTLV-2-infected PBMCs (Figure 1c).

Eight miRNAs were significantly altered in both infected cell populations, suggesting a potential signature related to HTLV-2 infection. Three were similarly upregulated (miR-125a-3p, miR-381-3p, and miR-502-5p) in both samples, while five miRNAs (miR-708-5p, miR-548d-5p, miR-548c-5p, miR-1-3p, and miR-511-5p) were differentially modulated in infected PBMCs and BJABGu (upregulated and downregulated, respectively).

### 2.2. Exploring miRNA Target Genes

**Figure 2:**
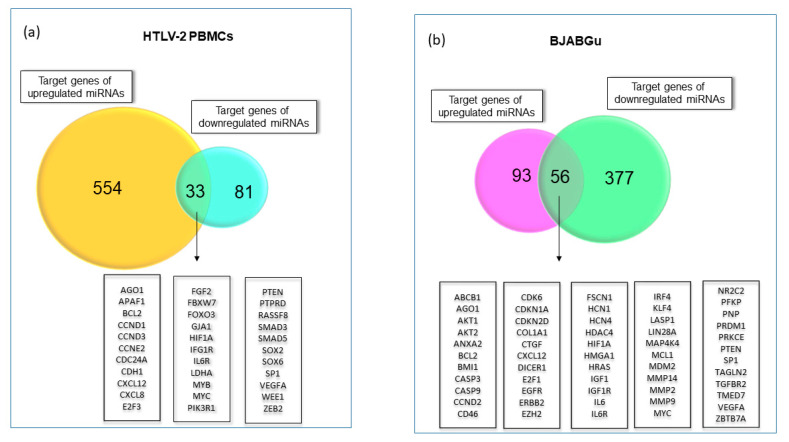
Target genes of deregulated miRNAs. The Venn diagram indicates the target genes of differentially expressed (down or upregulated) in HTLV-2-infected PBMCs (**a**) and BJABGu (**b**). The target genes that are common between the two groups are listed in the boxes.

We focused on miRNAs that were more considerably [log10 (FC) > |1|] modulated in infected PBMCs and BJABGu cell lines. Then, their corresponding validated target genes were identified by examining the miRTarBase.2020 database. To achieve more consistent results, we only considered the target genes that were validated by strong experimental evidence, such as being functional and non-functional, which was used as the first selection criterion. In addition, after performing the gene ontology (GO) analysis, we filtered out miRNAs whose target genes were related to non-statistically significant GO terms in more than one category. Figure 2 represents the number of related target genes after filtering out overlapping target genes. Infected HTLV-2 PBMCs showed a large number of upregulated genes (554), while BJABGu exhibited a higher number of downregulated targets (377) (Figure 2a,b). The intersection of target mRNAs for downregulated and upregulated miRNAs showed a total of 33 and 56 genes attributed to infected PBMCs and BJABGu, respectively. Furthermore, by comparing these two gene lists, eight target genes were shared by both cell models (AGO1, CXCL12, IGF1R, IL6R, MYC, PTEN, SP1, and VEGFA).

With regard to miRNAs belonging to the HTLV-2 putative signature, miR-548c-5p did not have validated targets, while the other four (miR-511-5p, miR-381-3p, miR-502-5p, and 548d-5p) did not meet inclusion criteria related to GO categories. Therefore, only the target genes of miR-125a-3p, miR-708-5p, and miR-1-3p were considered for further analyses.

### 2.3. Top Enriched Pathways Related to miRNAs Affected by HTLV-2 Were Driven by Mediators of Cell Activation and Differentiation

Potential cellular mechanisms affected by miRNAs with [log10 fold change (FC) > |1|] were found by querying both KEGG and Reactome databases through separate analyses of the target genes of both up regulated and downregulated miRNAs. Separate enrichment analyses of differentially expressed miRNA target genes have been demonstrated to identify the pathways that might more accurately reflect phenotypic differences [41].

**Figure 3:**
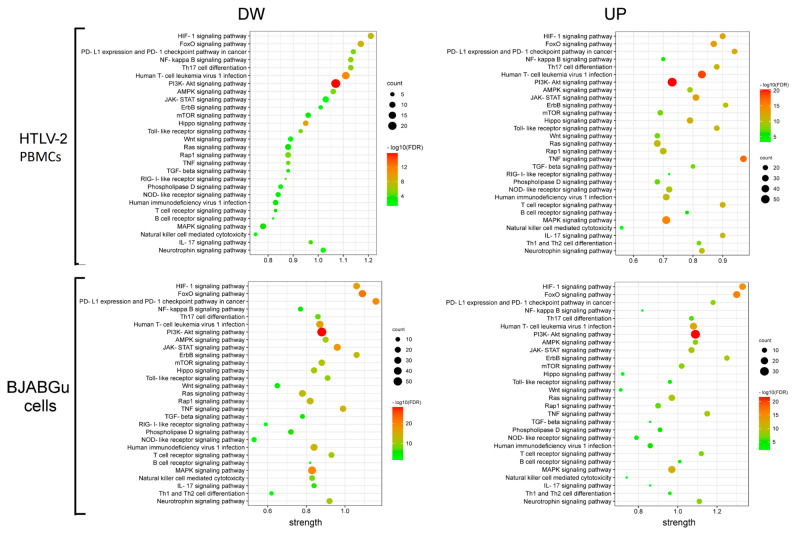
Bubble plot of the KEGG pathway enrichment of the target genes. The strength of the enriched signaling pathways associated with the target genes of the downregulated (DW) miRNAs (**left panel**) and upregulated (UP) miRNAs (**right panel**) in HTLV-2 infected PBMCs (**upper panel**) and BJABGu cells (**bottom panel**). The size and color of each bubble represent the number of target genes in each pathway and its corresponding *p*-value, respectively.

Firstly, we investigated the functional aspects of the target genes through KEGG analysis and reviewed the enriched terms (FDR < 0.001), focusing on those referring to retrovirus infection, signaling pathways, and immune cell maturation (Figure 3). The analysis revealed that all targeted gene panels shared several selected KEGG terms, including HTLV-1 and HIV-1 infections; Th17 differentiation; and signaling pathways regulated by PD-L1/PD-1, PI3k–Akt, p53, NF-kB, FoxO, and HIF-1. In this regard, considering the number of genes enriching each category, in combination with the significance level and the fold enrichment fold (strength), PI3k–Akt signaling seemed to be a key target pathway among the crucial ones affected by miRNA alteration in both HTLV-2-infected samples. Similarly, Th17 differentiation was found to be significantly overrepresented in all groups. Interestingly, the cytokine signaling pathway was the only one enriched in all target gene sets was that driven by IL-17.

To gain additional insight into the activators, regulators, and adaptor molecules and their correlation with the cellular pathways affected by HTLV-2-deregulated miRNAs, we performed a Reactome enrichment analysis. The enrichment of pathways, including the interleukins and PI3k–Akt network, as well as the RUNXs family members, was confirmed (Table 1). Interestingly, the results of the HTLV-2-infected PBMC and BJABGu cells enrichment analyses showed differences between the cell types. Among the interleukins, IL-1, IFN-γ, and IL-17 were significantly overrepresented only in the infected PBMCs, while IL-2, TNF, and IL-10 were exclusively associated with BJABGu cells. PI3k activation by both NTRK3 and erythropoietin was found only in the BJABGu cells. Different pathways were found to be associated with RUNX3 modulation between infected PBMCs and BJABGu.

By applying gene enrichment analysis (GEA) to the target genes of miRNAs belonging to the signature (Table 2), we observed that miR-125a-3p was associated with Th17 differentiation and signalling pathways driven by IL-4 and IL-13. Additionally, miR-1-3p and miR-708-5p were correlated with RUNX3 and RUNX2, respectively.

### 2.4. RUNX2 Is Deregulated in Presence of Tax Proteins

**Figure 4:**
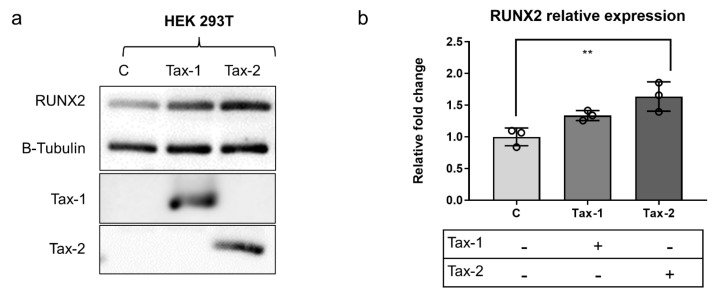
On the left (**a**), representative Western blot analysis of the HEK293T control cells and cells transfected with Tax-1 pJFE or Tax-2 pJFE showing an increase in RUNX2 protein expression in cells transfected with the HTLV-2 Tax protein. On the right (**b**), histogram bars represent the densitometric analysis of the Western blot bands corresponding to RUNX2 normalized to tubulin. ** = *p* value < 0.01 using one-way ANOVA; c = control non-transfected cells; Tax-1 = cells transfected with Tax-1 pJFE; Tax-2 = cells transfected with Tax-2 pJFE.

The reciprocal activation of RUNX2 and PI3k–Akt axis is known to cooperate in cell transformed and not-transformed proliferation [42]. Further, RUNX2 has been demonstrated to play a role in T-cell lymphoma, acute myeloid leukemia, and multiple myeloma and to facilitate the response to viral infection through the control of Interferon Regulatory Factor 7 (IRF7) [43,44,45]. Despite this evidence, RUNX2 has never been studied in the context of HTLV infection, until now. Based on the results of the enrichment analyses, which suggest PI3k–Akt signaling and RUNX transcription factors as putative targets of the HTLV deregulated miRNAs, we analyzed the RUNX2 protein expression in HEK293T cells transfected with Tax-1 or Tax-2 recombinant vectors. Interestingly, in presence of Tax-2 but not Tax-1, the RUNX2 protein was significantly more represented compared with the non-transfected cells (Figure 4). We performed qPCR to analyze the same transfected cell samples for the relative expression of miR-1-3p and miR-708-5p, which were found to be correlated with the RUNX-enriched pathway (Table 2). In agreement with previous studies [46], our analysis evidenced the expression of both miRNAs in HEK293T cells and their expression was modulated after the transfection of Tax-1 and Tax-2 (Supplementary Figure S2).

### 2.5. Multiple Overlapping Pathways in Different Single Node Networks Revealed Potential Strong Functional Relationships

**Figure 5:**
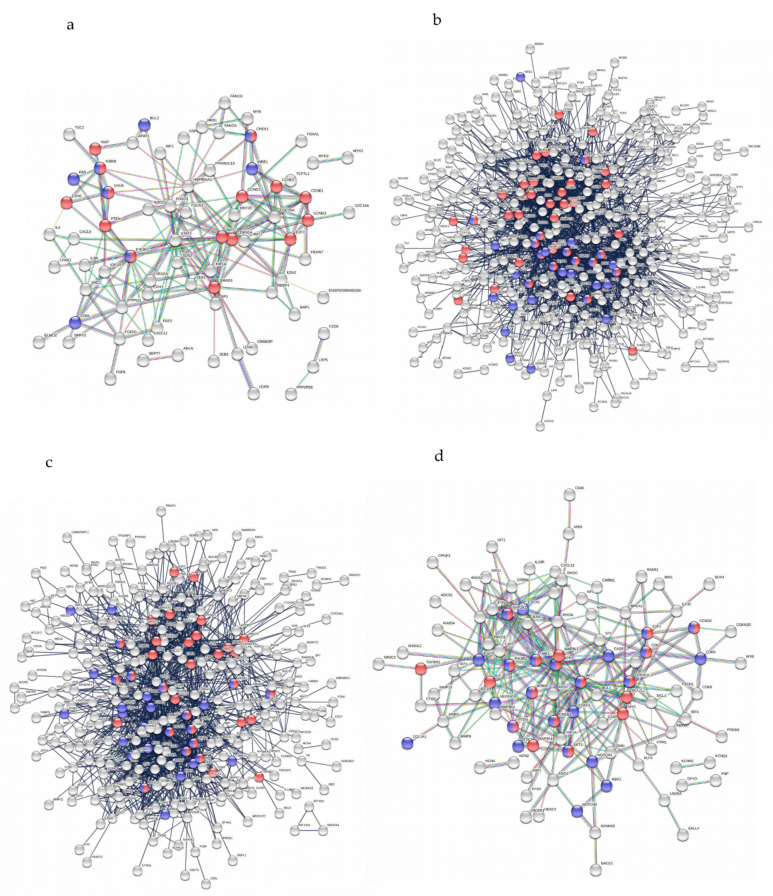
Analysis of PPIs of the target genes of miRNAs down regulated and upregulated in infected PBMCs ((**a**,**b**) respectively) and BJABGu cells ((**c**,**d**) respectively) related to HIV-1 and HTLV-1 annotations. Red nodes are associated with HTLV-1 infection, while blue nodes are associated with HIV-1 infection. Double coloured nodes share both annotations (genes name are listed in Supplementary S2).

Protein–protein interactions (PPIs) were mapped using the STRING v. 11 database by setting the confidence score to the highest value (0.900) in order to visualize the most specific and strengthened associations within each panel of miRNA target genes. As many of the genes targeted by modulated miRNAs were common in different pathways, corresponding proteins were found to be highly interconnected in a central module, as observed in all networks (Supplementary Figure S1 and Table S1), highlighting how processes triggered by virus replication may occur as a cascade of overlapping events. As HTLVs have been proven to interfere with HIV-1 replication in co-infected patients [15], the following analysis of PPIs was addressed to identify PPIs associated both with HTLV-1 and HIV-1 (Figure 5). HTLVs and HIV-1 infections resulted were correlated with genes common among different signalling pathways such as PI3k/AKT, MAPK, IKB, CDK, and NF-kB. Pathways related to co-receptor CXCR4 and caspases (i.e., CASP3/-8/-9) were only annotated with both HTLV-2 and HIV-1.

## 3. Discussion

Increasing evidence has demonstrated that retroviruses orchestrate cellular dynamics by altering miRNA expression and, in turn, miRNA target gene functionality, in an attempt to achieve successful replication [47].

Our study identified a potential HTLV-2-related miRNA signature composed of eight miRNAs whose expression was modified in both infected PBMCs and BJABGu cell lines. Additionally, this fact pointed out that the signature was independent of possible coinfecting pathogens in patients. Remarkably, three of the eight miRNAs have been proven to be involved in hindering the development of several cancers, such as thyroid carcinoma (miR-125a-3p), colorectal (miR-381-3p), and ovarian and bladder cancers (miR-502-5p) [48,49,50], suggesting that their modulation induced by the virus might contribute to hampering cell proliferation. We observed a general upregulation in the expression of miRNAs in HTLV-2-infected PBMCs. Since several studies have demonstrated HTLV-2 patients having a low activation status [51], we speculated about a potential correlation between miRNA upregulation and a low activation profile, in accordance with previous evidence of a global miRNAs downregulation upon T cell stimulation [52].

The KEGG and Reactome analyses of the selected altered miRNAs revealed that HTLV-2 infection may affect common biological mechanisms through modulation of cytokines/chemokines, transcriptional signaling pathways, and Th cell differentiation, including Th17 differentiation in particular. Recently, higher levels of Th17 cells, which are primarily located in the mucosal tissues and expressing HIV receptors and co-receptors (CD4, α4β7, CCR5, and CXCR4), were found to be positively correlated with more protective T-cell responses against HIV-1 [53]. Furthermore, Th17 CD4+ T cells are supposed to be long-living viral reservoirs in patients receiving antiretroviral therapy [54]. Taking into account these recent findings, our preliminary data may open up a new intriguing scenario for studying HTLV-2/HIV-1 co-infection as a model for discovering novel strategies to curb HIV-1 persistence.

Our evidence in favor of distinct PI3K/AKT/mTOR pathways in different cell types is in agreement with previous reports strengthening PI3ks role in affecting T and B cell differentiation in combination with different mediators [55], suggesting that HTLV-2 may adopt multiple strategies to prolong its own viral replication and delayed apoptosis. In this regard, both Tax-1 and Tax-2, in particular Tax-2b, have been shown to abrogate the suppressor activity of p53 in T cells [22]. Remarkably, while it has been found that the TGFβ signaling pathway is promoted by Tax-1 through Smad transcription factors, as well as by HBZ, there was no evidence of it being modulated to Tax-2 and APH-2 [56]. In contrast, our data led us to hypothesize a potential ability of HTLV-2 to affect TGF-β immunomodulatory signaling.

Besides the already mentioned transcription factors, compelling evidence of the RUNX-3- and RUNX-2-signalling pathway association with the HTLV-2 miRNA signature (in particular to miR-1-3p and miR-708-5p, respectively), has pointed out the potential involvement of RUNXs in achieving successful HTLV-2 replication. Accumulating previous data may suggest possible explanations for the contribution of RUNXs contribution. Activating RUNX-3 signaling has been demonstrated to cooperate in the function of cytotoxic T cells [57]. Similarly, RUNX-2 has been proven to be critical for the persistence of T cell mediated responses (i.e., CD8+ memory T cells) during chronic viral infection [58,59]. The enrichment pathway results shown in the present study are supported by previously reported data demonstrating that miR-1-3p and miR-708-5p are involved in the activation of the PI3k–Akt signaling pathway and that the RUNX2 and PI3k–Akt axis participate reciprocally in their activation [42,60,61]. The overexpression of RUNX2 has been reported in several tumors, including T-cell lymphoma, acute myeloid leukemia [43,44], and, recently, T-cell acute lymphoblastic leukemia (T-ALL) [62]. In mouse models, upregulation of Runx2 has also been shown to induce the repression of myeloid differentiation [44]. The pivotal role of RUNX2 expression in T-cell lineage development has also been demonstrated by the ectopic expression of RUNX2, which caused significant deregulation of thymocyte maturation and an increase in the expression of immature CD8 cells [63].

MiRNAs deregulated by HTLV-1 have been identified in recently published studies [5,28,35,36,37,38,39]. Interestingly, none of the eight miRNAs identified in our study were described to be deregulated in the presence of HTLV-1 or in subjects with ATL. Furthermore, the fact that we found three out of eight miRNAs altered by HTLV-2 infection in both T and B cell populations made us more confident in the consistency of our findings. The differences in miRNA expression in the two infected cell populations might mainly be attributed to their different activation status. Future analysis and validation will be required to address the regulatory mechanisms and biological effects of these miRNAs. This study is limited to a small number of subjects, but represents the first contribution to define the effect of HTLV-2 infection on the aberrant expression of miRNAs and their molecular role in viral persistence and coinfection.

## 4. Materials and Methods

### 4.1. Primary and Immortalized Cells

Six Italian HTLV-2 mono-infected individuals and six healthy volunteer donors were recruited at ASMN of Reggio Emilia, Italy. Infected patients belonged to a cohort of (Caucasian; 5 male and 1 woman; with a mean (SD) age of 49.7 (5.2)) HIV-1 seronegative subjects with high-risk behaviors, drug abuse, and unprotected sexual intercourse. The uninfected subject group was matched by sex, age, and ethnicity. The infected subjects were diagnosed as HTLV-2 seropositive in 2005 and were followed up over the years to monitor the occurrence of clinical symptomatology and virological parameters. The enrolled infected patients harbored HTLV-2 subtype 2b, as demonstrated by sequencing of the long terminal repeat region of the viral isolates. These six infected patients were selected for study because of their high proviral load, ranging from 2994 cp/10^5^ cells (mean proviral load of 4183 cp/10^5^ cells) at the time of enrolment. The BJABGu was established by growing the HTLV-2-Gu isolate [62] in the Epstein–Barr-negative B cell line BJAB [51] and was found to harbor 1.8 copies of provirus per cell by determining the Tax-2 gene copies number through real-time PCR.

Blood samples from both HTLV-2 positive patients and controls were pooled separately. PBMCs were isolated from the two pools through Ficoll density gradient centrifugation and were stored in RNAlater at 80 °C or were directly processed for miRNA extraction using the mirVana miRNA isolation kit (Ambion, Austin, TX, USA).

This research was conducted according to the guidelines of the Declaration of Helsinki and was approved by the Provincial Ethics Committee of the Santa Maria Nuova Hospital and the Reggio Emilia Local Health Authority (Ref. number 0025318, 13 September 2010). Written informant consent was obtained from the subjects involved in the study.

The small sample size of participants was deemed appropriate because of the exploratory nature of this research and the low prevalence of HTLV-2 infection among HIV-1 negative subjects [33].

### 4.2. miRNA Expression Analysis

The miRNA expression was determined by applying the Megaplex Pools protocol (Applied Biosystems, Waltham, MA, USA) and using the Human Pool A contains RT primers for 377 unique microRNAs and 4 endogenous controls. In total, 100 ng RNA/samples were converted into cDNA using the TaqMan^®^ MicroRNA Reverse Transcription Kit and the Megaplex™ RT Primers. The following RT reaction protocol was used: 40 cycles at 16 °C for 2 min, 42 °C for 1 min, and 50 °C for 1 s followed by 1 step at 85 °C for 5 min. The obtained cDNAs (2.5 μL/sample) were then subjected to PCR using a TaqMan PreAmp Master Mix kit and Megaplex PreAmp Primers Pool A (Applied Biosystems, Whaltam, MA, USA). The following thermal-cycling conditions were applied: denaturation for 10 min at 95 °C, 1 step at 55 °C for 2 min followed by 2 min at 72 °C, and 12 cycles (95 °C for 15 s, 60 °C for 4 min) to synthesize single-stranded cDNA from the total RNA samples. The preamplified cDNA products were added to TaqMan Universal PCR Master Mix, No AmpErase UNG, and were loaded onto the TaqMan MicroRNA Array A for PCR amplification using the following protocol: 50 °C for 2 min, 94.5 °C for 10 min, and 40 cycles at 97 °C for 30 s and 59.7 °C for 1 min. All of the reactions mentioned above were conducted on a 7900HT Fast Real-Time PCR system (Applied Biosystems, Whaltam, MA, USA).

Data analysis was performed using Relative Quantification Manager 1.2 software (Applied Biosystems) and comparative Ct method (ΔΔCt). The amplification signal was checked for each sample using SDS Version 2.3 software (Applied Biosystems, Whaltam, MA, USA). Undetermined raw Ct values were set to 40. The RNA extracted from the pool of healthy donors was used as the calibrator, whereas the small noncoding MammU6 RNA was tested as the housekeeping gene.

Hierarchical clustering of the samples was obtained by loading the miRNA fold—change expression on http://www.heatmapper.ca (accessed on 1 June 2022) [63] and by applying ‘Average Linkage’ and ‘Euclidean’ as the clustering and distance methods, respectively.

### 4.3. Functional Analysis of miRNAs Target Genes

MiRNA targets were extracted from miRTarBase v. 9 released on 15 September 2021. We only filtered out validated targets that were annotated with miRNA–target interactions (MTIs), supported by strong experimental evidence, such as those obtained by Report Assay, Western blot, pPCR, and both Report Assay and Western blot.

GO annotation data were obtained using the Gene Ontology website (http://geneontology.org/ (accessed on 1 June 2022)).

Pathway annotations were provided by Reactome (version 13 October 2021) and KEGG (version 1 October 2021) databases [64]. Only enriched terms with a significant *p* value less than 0.05 as a statistical threshold were retained for functional consideration. Interacting proteins were further found out using the STRING (version 11.5) tool to perform the PPI network analysis. A confidence score of 0.900 (the highest) was set as a threshold.

### 4.4. Cell Lines and Transfection

HEK293T cells were maintained in Dulbecco’s modified Eagle’s Medium (DMEM) supplemented with 10% fetal calf serum (FCS), L-glutamine (2 mM), and Penicillin G (100 U/L)/Streptomycin (100 mcg/L). Cells were grown at 37 °C in a humidified atmosphere with 5% CO_2_. For analysis of the protein content, 4.5 × 10^5^ cells were seeded in six-well plates and, after 24 h, transfected using TransIT-LT1 transfection reagent (cat. MIR2300, Mirus Bio LLC, Madison, WI, USA), following manufacturer’s protocol.

### 4.5. Plasmids

HEK293T cells were transfected with 1 µg of pJFE-Tax-1, pJFE-Tax-2 (both previously described in [28] or mock-transfected with the empty pSGM vector.

### 4.6. Protein Extraction

HEK293T cells were harvested 24 h after transfection with 1 µg of each vector. Cells were washed two times using ice-cold PBS and then lysed using RIPA buffer (50 mM Tris HCl pH 7.5, 150 mM NaCl, 1% NP-40, 0.5% sodium deoxycholate, 0.1% SDS) supplemented with a protease inhibitors cocktail. The lysates were then incubated in ice for 30 min, frozen at −80 °C for at least 1 h, and then centrifuged for 30 min at 14,000 rpm at 4 °C.

### 4.7. Western Blotting

The total protein concentration in the cell lysates was determined by Bradford Coomassie brilliant blue assay (Sigma-Aldrich, St. Louis, MO, USA). Equal amounts of cellular proteins were resolved in SDS polyacrylamide gel electrophoresis (SDS-PAGE) and transferred to a PVDF membrane (Cytiva life sciences, Marlborough, MA, USA). Membranes were first saturated in TBS solution containing 5% non-fat milk and 0.1% Tween20, and then incubated with specific primary and secondary antibodies. Anti-β-tubulin was used as a loading control. Bound antibodies were revealed using WesternBrightTM ECL (cat. K-12045-D50, Advansta, San Jose, CA, USA), according to the manufacturer’s instructions. Densitometry analysis of the Western blot protein bands was performed using the Fiji-ImageJ software [65] and statistical analysis was performed through ordinary one-way ANOVA using GraphPad Prism 7 software (GraphPad Inc., La Jolla, CA, USA).

### 4.8. Antibodies

Rabbit monoclonal anti-RUNX2 (1:1000, cat. 8486, Cell Signaling, Danvers, MA, USA), rabbit polyclonal anti-β Tubulin (1:400, cat. sc-9104, Santa Cruz, Santa Cruz, CA, USA), mouse monoclonal anti-Tax-1 derived from hybridoma 168-A51 (1:2, AIDS research and Reagent Program, National Institutes of Health), and rabbit polyclonal anti-Tax-2 (1:1000, previously described in [66]) were used as primary antibodies for Western blotting. Horseradish peroxidase-conjugated anti-rabbit (1:10,000 cat. 31460, Thermo Fisher Scientific, Waltham, MA, USA) and anti-mouse (1:10,000, cat. 31430, Thermo Fisher Scientific, Waltham, MA, USA) were used as secondary antibodies.

### 4.9. RT-qPCR Analysis

RNA was reverse transcribed using the TaqMan^TM^ Advanced miRNA cDNA Synthesis Kit (cat. A28007, Applied Biosystems, Waltham, MA, USA) according to the manufacturer’s instructions. RT-qPCR reactions were performed using TaqMan™ Fast Advanced Master Mix (cat. 4444557, Applied Biosystems, Waltham, MA, USA), as described by the manufacturer’s instructions. Specific miRNAs probes (cat. A25576, Applied Biosystems, Waltham, MA, USA) were applied for the expression level analysis of miR- miR-1-3p and miR-708-5p. The qPCR was performed using a CFX Connect Real-Time PCR System (Bio-Rad Laboratories, Hercules, CA, USA). The samples were incubated in a 96-well plate at 95 °C for 20 s, followed by 40 cycles of 95 °C for 1 s and 60 °C for 20 s. The gene expression was normalized to the mean of the reference miRNA miR-195-5p to be used as the reference miRNA and as the endogenous control to quantify miRNAs [67]. Relative quantification of the miRNA expression was calculated according to the ΔΔCt method [68]. Each measurement was carried out in triplicate in three different experiments. Differences in the relative expression levels were analyzed via ordinary one-way ANOVA test using GraphPad Prism 7 software (GraphPad Inc., La Jolla, CA, USA).

## Figures and Tables

**Table 1:** Comparative analysis of selected signaling pathways gathered from the Reactome source.

Reactome Terms	Down miRNAs in Infected PBMCs	Up miRNAs in Infected PBMCs	Down miRNAs in BjabGu	Up miRNAs in BjabGu
Regulation of RUNX1 Expression and Activity	1.61 *; 0.0018 **	1.14 *; 0.00055 **	1.21 *; 0.0009 **	n.d. ^‡^
Transcriptional regulation by RUNX2	n.d	0.83 *; 1.89 × 10^−9^ **	0.89 *; 9.55 × 10^−9^ **	0.9 *; 0.008 *
Transcriptional regulation by RUNX3	1.11 *; 0.00067 **	0.73 *; 0.0001 **	0.8 *; 8.8 × 10^−5^ **	1 *; 0.0023 **
RUNX2 regulates genes involved in cell migration	n.d.	n.d.	1.45 *; 0.00066 **	n.d.
RUNX3 regulates WNT signaling	1.81 *; 0.0071 **	1.32 *; 0.0025 **	n.d.	n.d.
RUNX3 regulates CDKN1A transcription	n.d.	n.d	1.41 *; 0.0057 **	n.d
RUNX3 regulates p14-ARF	n.d.	n.d.	1.31 *; 0.0103 **	n.d.
Interleukin-6 signaling	1.67 *; 0.0133 **	1.18 *; 0.0068 **	1.32 *; 0.0018 **	1.56 *; 0.0226 **
Interleukin-4 and Interleukin-13 signaling	1.44 *; 4.86 × 10^−15^ **	1.08 *; 1.7 × 10^−22^ **	1.13 *; 1.25 × 10^−20^ **	1.37 *; 8.07 × 10^−16^ **
Interleukin-37 signaling	1.39 *; 0.0487 **	n.d.	n.d.	n.d.
Interleukin-3, Interleukin-5 and GM-CSF signaling	1.16 *; 0.0372 **	n.d.	0.82 *; 0.0128 **	n.d.
Interleukin-1 family signaling	0.88 *; 0.0296 **	n.d.	n.d.	n.d.
Regulation of IFNG signaling	n.d.	1.08 *; 0.0143 **	n.d.	n.d.
Interleukin-17 signaling	n.d.	0.75 *; 0.00066 **	n.d.	n.d.
Interleukin-2 family signaling	n.d.	n.d.	0.87 *; 0.0078 **	n.d.
TNF signaling	n.d.	n.d.	0.79 *; 0.04 **	n.d.
Interleukin-10 signaling	n.d.	n.d.	0.78 *; 0.0439 **	n.d.
Interleukin-12 signaling	n.d.	n.d.	n.d.	1.16 *; 0.0075 **
PI3k cascade: FGFR1	1.61 *; 0.00018 **	n.d.	n.d.	n.d.
PI3k cascade: FGFR3	1.58 *; 0.0021 **	n.d.	n.d.	n.d.
PI3k cascade: FGFR4	1.54 *; 0.0029 **	0.92 *; 0.041 **	n.d.	n.d.
PI3k cascade: FGFR2	1.47 *; 0.0044 **	n.d.	n.d.	n.d.
PI3k events in ERBB2 signaling	n.d.	1.02 *; 0.0211 **	1.06 *; 0.0443 **	1.52 *; 0.0028 **
AKT-mediated inactivation of FOXO1A		1.4 *; 0.0461 **	1.53 *; 0.0217 **	2 *; 0.0029 **
Negative regulation of the PI3K/AKT network	1.03 *; 0.0018 **	0.87 *; 1.28 × 10^−10^ **	0.93 *; 8.3 × 10^−10^ **	1.07 *; 1.58 × 10^−5^ **
PI5P, PP2A and IER3 Regulate PI3K/AKT Signaling	0.99 *; 0.0099 **	0.83 *; 4.31 × 10^−8^ **	0.89 *; 7.46 × 10^−8^ **	1.01 *; 0.00057 **
CD28 dependent PI3K/Akt signaling	n.d.	1.14 *; 3.68 × 10^−5^ **	1.09 *; 0.0026 **	1.48 *; 0.00045 **
Activated NTRK3 signals through PI3K	n.d.	n.d.	1.36 *; 0.0439 **	n.d.
Erythropoietin activates Phosphoinositide-3-kinase (PI3K)	n.d.	n.d.	1.42 *; 1.87 × 10^−5^ **	n.d.
AKT phosphorylates targets in the cytosol	n.d.	n.d.	1.29 *; 0.00043 **	1.58 *; 0.0019 **
AKT phosphorylates targets in the nucleus	n.d.	n.d.	n.d.	1.73 *; 0.00072 **

* Fold enrichment, ** FDR *p*-value, ^‡^ not determined

**Table 2:** Pathways enriched in the gene list of HTLV-2-related miRNA signature.

miRNAs	HTLV-2+ vs. Uninfected	BJABGu vs. BJAB	Targets	KEGG GEA	Reactome GEA
125a-3p	**2.07 (FE)**	**2.59 (FE)**	GPC4, MTA1, PRDM1, RHOA, FYN, IL23R, GIT1, VEGFA, IRF4, XBP1, EZH2, BRCA1, IL6, NRG1	EGFR tyrosine kinase inhibitor resistance (1.73 *; 0.0055 **) Inflammatory bowel disease (1.67; 0.0497) Th17 cell differentiation (1.62; 0.0077)	ERBB2 signaling (1.92; 0.0387) IL-4 and IL-13 signaling (1.72; 0.0163) Interleukins signaling (1.2; 0.0435)
708-5p	**1.04 (FE)**	**−3.13 (FE)**	BMI1, ZEB2, BIRC5, AKT2, CD44, TMEM88, EYA3, NNAT, AKT1, CCND1, MMP2, EZH2, PARP1, BCL2, CASP2, CD274, CNTFR, SMAD3, IKBKG, KDM1A	Pancreatic cancer (1.83; 1.8 × 10^−6^) Chronic myeloid leukemia (1.81; 1.8 × 10^−6^) AGE-RAGE signaling pathway in diabetic complications (1.78; 3.21 × 10^−7^)	AKT-mediated inactivation of FOXO1A (2.69; 0.0161) RUNX2 regulates genes involved in cell migration (2.39; 0.0362) Activation of BAD and translocation to mitochondria (2.29; 0.0016)
1-3p	**2.07 (FE)**	**−2.36 (FE)**	CEBPA, MEF2A, GATA4, HCN4, HDAC4, FOXP1, HCN2, PTMA, PTBP1, MET, CAND1, ANXA2, HAND2, IGF1, TMSB4X, KCNJ2, GJA1, XPO6, POGK, TAGLN2, LASP1, ADAR, KCNE1, BDNF, G6PD, SOX6, ATP6V1B2, LARP4, CNN3, PNP, KIF2A, HSPD1, HSPA4, PIM1, CALM3, PPP2R5A, PAX3, TWF1, TWF2, FN1, NOTCH3, SLC8A1, EDN1, PRKCE, FABP3, SNAI2, SOX9, PGD, SRF, IL11, YWHAZ, CCND1, MYOCD, TKT, CCL2, CDK4, SP1, ETS1, FASN, PIK3CA, TH, MPL, API5, SPRED1, ASPH, ND1, COX1, FRS2, FZD7, AGO1, KRAS, NAIP, VEGFA, RARB, BAG4, ABCB1, TWIST1, TNKS2, CXCL12	Pentose phosphate pathway (1.41; 0.0039) AGE-RAGE signaling pathway in diabetic complications (1.4; 7.86 × 10^−9^) Bladder cancer (1.38; 0.00071)	RUNX3 regulates p14-ARF (1.92; 0.0123) Signaling by FGFR3 fusions in cancer (1.87; 0.0126) Signaling by FGFR4 in disease (1.83; 0.0128)

* fold enrichment, ** FDR *p*-value.

## Data Availability

The data that support the findings of this study are available from the corresponding author upon request.

## Supplementary Materials

The following supporting information can be downloaded at: https://www.mdpi.com/article/10.3390/ijms23147583/s1.

## References

[1] O’Brien J., Hayder H., Zayed Y., Peng C. (2018). Overview of MicroRNA Biogenesis, Mechanisms of Actions, and Circulation. Front. Endocrinol..

[2] Roberts A.P.E., Lewis A.P., Jopling C.L. (2011). The Role of MicroRNAs in Viral Infection. Prog. Mol. Biol. Transl. Sci..

[3] Vojtechova Z., Tachezy R. (2018). The Role of MiRNAs in Virus-Mediated Oncogenesis. Int. J. Mol. Sci..

[4] Balasubramaniam M., Pandhare J., Dash C. (2018). Are MicroRNAs Important Players in HIV-1 Infection? An Update. Viruses.

[5] Sampey G.C., Van Duyne R., Currer R., Das R., Narayanan A., Kashanchi F. (2012). Complex Role of MicroRNAs in HTLV-1 Infections. Front. Genet..

[6] Sanghvi V.R., Steel L.F. (2012). RNA Silencing as a Cellular Defense against HIV-1 Infection: Progress and Issues. FASEB J. Off. Publ. Fed. Am. Soc. Exp. Biol..

[7] Houzet L., Jeang K.-T. (2011). MicroRNAs and Human Retroviruses. Biochim. Biophys. Acta.

[8] Rezaie J., Aslan C., Ahmadi M., Zolbanin N.M., Kashanchi F., Jafari R. (2021). The Versatile Role of Exosomes in Human Retroviral Infections: From Immunopathogenesis to Clinical Application. Cell Biosci..

[9] Lairmore M.D., Franchini G. (2007). Human T-Cell Leukemia Virus Types I and II. Fields Virology.

[10] Mahieux R., Gessain A. (2011). HTLV-3/STLV-3 and HTLV-4 Viruses: Discovery, Epidemiology, Serology and Molecular Aspects. Viruses.

[11] Pique C., Jones K.S. (2012). Pathways of Cell-Cell Transmission of HTLV-1. Front. Microbiol..

[12] Ciminale V., Rende F., Bertazzoni U., Romanelli M.G. (2014). HTLV-1 and HTLV-2: Highly Similar Viruses with Distinct Oncogenic Properties. Front. Microbiol..

[13] Iwanaga M., Watanabe T., Yamaguchi K. (2012). Adult T-Cell Leukemia: A Review of Epidemiological Evidence. Front. Microbiol..

[14] Bertazzoni U., Ciminale V., Romanelli M.G. (2018). Editorial: Molecular Pathology of HTLV-1. Front. Microbiol..

[15] Pilotti E., Bianchi M.V., De Maria A., Bozzano F., Romanelli M.G., Bertazzoni U., Casoli C. (2013). HTLV-1/-2 and HIV-1 Co-Infections: Retroviral Interference on Host Immune Status. Front. Microbiol..

[16] Hjelle B., Appenzeller O., Mills R., Alexander S., Torrez-Martinez N., Jahnke R., Ross G. (1992). Chronic Neurodegenerative Disease Associated with HTLV-II Infection. Lancet Lond. Engl..

[17] Casoli C., Pilotti E., Bertazzoni U. (2007). Molecular and Cellular Interactions of HIV-1/HTLV Coinfection and Impact on AIDS Progression. AIDS Rev..

[18] Beilke M.A., Theall K.P., O’Brien M., Clayton J.L., Benjamin S.M., Winsor E.L., Kissinger P.J. (2004). Clinical Outcomes and Disease Progression among Patients Coinfected with HIV and Human T Lymphotropic Virus Types 1 and 2. Clin. Infect. Dis. Off. Publ. Infect. Dis. Soc. Am..

[19] Melamed A., Witkover A.D., Laydon D.J., Brown R., Ladell K., Miners K., Rowan A.G., Gormley N., Price D.A., Taylor G.P. (2014). Clonality of HTLV-2 in Natural Infection. PLoS Pathog..

[20] Forlani G., Shallak M., Accolla R.S., Romanelli M.G. (2021). HTLV-1 Infection and Pathogenesis: New Insights from Cellular and Animal Models. Int. J. Mol. Sci..

[21] Heym S., Mohr C.F., Engelbrecht H.C., Fleckenstein B., Thoma-Kress A.K. (2022). Alternative NF-ΚB Signaling Discriminates Induction of the Tumor Marker Fascin by the Viral Oncoproteins Tax-1 and Tax-2 of Human T-Cell Leukemia Viruses. Cancers.

[22] Martinez M.P., Al-Saleem J., Green P.L. (2019). Comparative Virology of HTLV-1 and HTLV-2. Retrovirology.

[23] Millen S., Meretuk L., Göttlicher T., Schmitt S., Fleckenstein B., Thoma-Kress A.K. (2020). A Novel Positive Feedback-Loop between the HTLV-1 Oncoprotein Tax and NF-ΚB Activity in T-Cells. Retrovirology.

[24] Romanelli M.G., Diani E., Bergamo E., Casoli C., Ciminale V., Bex F., Bertazzoni U. (2013). Highlights on Distinctive Structural and Functional Properties of HTLV Tax Proteins. Front. Microbiol..

[25] Shirinian M., Kfoury Y., Dassouki Z., El-Hajj H., Bazarbachi A. (2013). Tax-1 and Tax-2 Similarities and Differences: Focus on Post-Translational Modifications and NF-ΚB Activation. Front. Microbiol..

[26] Avesani F., Romanelli M.G., Turci M., Di Gennaro G., Sampaio C., Bidoia C., Bertazzoni U., Bex F. (2010). Association of HTLV Tax Proteins with TAK1-Binding Protein 2 and RelA in Calreticulin-Containing Cytoplasmic Structures Participates in Tax-Mediated NF-ΚB Activation. Virology.

[27] Fochi S., Mutascio S., Bertazzoni U., Zipeto D., Romanelli M.G. (2018). HTLV Deregulation of the NF-ΚB Pathway: An Update on Tax and Antisense Proteins Role. Front. Microbiol..

[28] Fochi S., Ciminale V., Trabetti E., Bertazzoni U., D’Agostino D.M., Zipeto D., Romanelli M.G. (2019). NF-ΚB and MicroRNA Deregulation Mediated by HTLV-1 Tax and HBZ. Pathogens.

[29] Higuchi M., Tsubata C., Kondo R., Yoshida S., Takahashi M., Oie M., Tanaka Y., Mahieux R., Matsuoka M., Fujii M. (2007). Cooperation of NF-KappaB2/P100 Activation and the PDZ Domain Binding Motif Signal in Human T-Cell Leukemia Virus Type 1 (HTLV-1) Tax1 but Not HTLV-2 Tax2 Is Crucial for Interleukin-2-Independent Growth Transformation of a T-Cell Line. J. Virol..

[30] Sheehy N., Lillis L., Watters K., Lewis M., Gautier V., Hall W. (2006). Functional Analysis of Human T Lymphotropic Virus Type 2 Tax Proteins. Retrovirology.

[31] Bertazzoni U., Turci M., Avesani F., Di Gennaro G., Bidoia C., Romanelli M.G. (2011). Intracellular Localization and Cellular Factors Interaction of HTLV-1 and HTLV-2 Tax Proteins: Similarities and Functional Differences. Viruses.

[32] Meertens L., Chevalier S., Weil R., Gessain A., Mahieux R. (2004). A 10-Amino Acid Domain within Human T-Cell Leukemia Virus Type 1 and Type 2 Tax Protein Sequences Is Responsible for Their Divergent Subcellular Distribution. J. Biol. Chem..

[33] Turci M., Romanelli M.G., Lorenzi P., Righi P., Bertazzoni U. (2006). Localization of Human T-Cell Lymphotropic Virus Type II Tax Protein Is Dependent upon a Nuclear Localization Determinant in the N-Terminal Region. Gene.

[34] Lewis M.J., Sheehy N., Salemi M., VanDamme A.-M., Hall W.W. (2002). Comparison of CREB- and NF-KappaB-Mediated Transactivation by Human T Lymphotropic Virus Type II (HTLV-II) and Type I (HTLV-I) Tax Proteins. Virology.

[35] Bellon M., Lepelletier Y., Hermine O., Nicot C. (2009). Deregulation of MicroRNA Involved in Hematopoiesis and the Immune Response in HTLV-I Adult T-Cell Leukemia. Blood.

[36] Moles R., Nicot C. (2015). The Emerging Role of MiRNAs in HTLV-1 Infection and ATLL Pathogenesis. Viruses.

[37] Ruggero K., Corradin A., Zanovello P., Amadori A., Bronte V., Ciminale V., D’Agostino D.M. (2010). Role of MicroRNAs in HTLV-1 Infection and Transformation. Mol. Aspects Med..

[38] Shadabi S., Delrish N., Norouzi M., Ehteshami M., Habibian-Sezavar F., Pourrezaei S., Madihi M., Ostadali M., Akhgar F., Shayeghpour A. (2021). Comprehensive High-Throughput Meta-Analysis of Differentially Expressed MicroRNAs in Transcriptomic Datasets Reveals Significant Disruption of MAPK/JNK Signal Transduction Pathway in Adult T-Cell Leukemia/Lymphoma. Infect. Agent. Cancer.

[39] Yamagishi M., Nakano K., Miyake A., Yamochi T., Kagami Y., Tsutsumi A., Matsuda Y., Sato-Otsubo A., Muto S., Utsunomiya A. (2012). Polycomb-Mediated Loss of MiR-31 Activates NIK-Dependent NF-ΚB Pathway in Adult T Cell Leukemia and Other Cancers. Cancer Cell.

[40] Bignami F., Pilotti E., Bertoncelli L., Ronzi P., Gulli M., Marmiroli N., Magnani G., Pinti M., Lopalco L., Mussini C. (2012). Stable Changes in CD4+ T Lymphocyte MiRNA Expression after Exposure to HIV-1. Blood.

[41] Hong G., Zhang W., Li H., Shen X., Guo Z. (2014). Separate Enrichment Analysis of Pathways for Up- and Downregulated Genes. J. R. Soc. Interface.

[42] Cohen-Solal K.A., Boregowda R.K., Lasfar A. (2015). RUNX2 and the PI3K/AKT Axis Reciprocal Activation as a Driving Force for Tumor Progression. Mol. Cancer.

[43] Blyth K., Vaillant F., Hanlon L., Mackay N., Bell M., Jenkins A., Neil J.C., Cameron E.R. (2006). Runx2 and MYC Collaborate in Lymphoma Development by Suppressing Apoptotic and Growth Arrest Pathways In Vivo. Cancer Res..

[44] Kuo Y.-H., Zaidi S.K., Gornostaeva S., Komori T., Stein G.S., Castilla L.H. (2009). Runx2 Induces Acute Myeloid Leukemia in Cooperation with Cbfbeta-SMMHC in Mice. Blood.

[45] Chopin M., Preston S.P., Lun A.T.L., Tellier J., Smyth G.K., Pellegrini M., Belz G.T., Corcoran L.M., Visvader J.E., Wu L. (2016). RUNX2 Mediates Plasmacytoid Dendritic Cell Egress from the Bone Marrow and Controls Viral Immunity. Cell Rep..

[46] Tian W., Dong X., Liu X., Wang G., Dong Z., Shen W., Zheng G., Lu J., Chen J., Wang Y. (2012). High-Throughput Functional MicroRNAs Profiling by Recombinant AAV-Based MicroRNA Sensor Arrays. PLoS ONE.

[47] Bernier A., Sagan S.M. (2018). The Diverse Roles of MicroRNAs at the Host–Virus Interface. Viruses.

[48] Song M., Wang N., Li Z., Zhang Y., Zheng Y., Yi P., Chen J. (2020). MiR-125a-3p Suppresses the Growth and Progression of Papillary Thyroid Carcinoma Cell by Targeting MMP11. J. Cell. Biochem..

[49] Ying Y., Li J., Xie H., Yan H., Jin K., He L., Ma X., Wu J., Xu X., Fang J. (2020). CCND1, NOP14 and DNMT3B Are Involved in MiR-502-5p-Mediated Inhibition of Cell Migration and Proliferation in Bladder Cancer. Cell Prolif..

[50] Zhan L., Yang J., Liu Y., Cheng Y., Liu H. (2021). MicroRNA MiR-502-5p Inhibits Ovarian Cancer Genesis by Downregulation of GINS Complex Subunit 2. Bioengineered.

[51] Bovolenta C., Pilotti E., Mauri M., Turci M., Ciancianaini P., Fisicaro P., Bertazzoni U., Poli G., Casoli C. (2002). Human T-Cell Leukemia Virus Type 2 Induces Survival and Proliferation of CD34(+) TF-1 Cells through Activation of STAT1 and STAT5 by Secretion of Interferon-Gamma and Granulocyte Macrophage-Colony-Stimulating Factor. Blood.

[52] Rodríguez-Galán A., Fernández-Messina L., Sánchez-Madrid F. (2018). Control of Immunoregulatory Molecules by MiRNAs in T Cell Activation. Front. Immunol..

[53] Falivene J., Ghiglione Y., Laufer N., Socías M.E., Holgado M.P., Ruiz M.J., Maeto C., Figueroa M.I., Giavedoni L.D., Cahn P. (2015). Th17 and Th17/Treg Ratio at Early HIV Infection Associate with Protective HIV-Specific CD8(+) T-Cell Responses and Disease Progression. Sci. Rep..

[54] Renault C., Veyrenche N., Mennechet F., Bedin A.-S., Routy J.-P., Van de Perre P., Reynes J., Tuaillon E. (2022). Th17 CD4+ T-Cell as a Preferential Target for HIV Reservoirs. Front. Immunol..

[55] Okkenhaug K., Turner M., Gold M.R. (2014). PI3K Signaling in B Cell and T Cell Biology. Front. Immunol..

[56] Panfil A.R., Dissinger N.J., Howard C.M., Murphy B.M., Landes K., Fernandez S.A., Green P.L. (2016). Functional Comparison of HBZ and the Related APH-2 Protein Provides Insight into Human T-Cell Leukemia Virus Type 1 Pathogenesis. J. Virol..

[57] Gülich A.F., Rica R., Tizian C., Viczenczova C., Khamina K., Faux T., Hainberger D., Penz T., Bosselut R., Bock C. (2021). Complex Interplay Between MAZR and Runx3 Regulates the Generation of Cytotoxic T Lymphocyte and Memory T Cells. Front. Immunol..

[58] Jadhav R.R., Im S.J., Hu B., Hashimoto M., Li P., Lin J.-X., Leonard W.J., Greenleaf W.J., Ahmed R., Goronzy J.J. (2019). Epigenetic Signature of PD-1+ TCF1+ CD8 T Cells That Act as Resource Cells during Chronic Viral Infection and Respond to PD-1 Blockade. Proc. Natl. Acad. Sci. USA.

[59] Olesin E., Nayar R., Saikumar-Lakshmi P., Berg L.J. (2018). The Transcription Factor Runx2 Is Required for Long-Term Persistence of Antiviral CD8+ Memory T Cells. ImmunoHorizons.

[60] Zhao Z., Qin X. (2020). MicroRNA-708 Targeting ZNF549 Regulates Colon Adenocarcinoma Development through PI3K/AKt Pathway. Sci. Rep..

[61] Ma Y., Zhou G., Li M., Hu D., Zhang L., Liu P., Lin K. (2018). Long Noncoding RNA DANCR Mediates Cisplatin Resistance in Glioma Cells via Activating AXL/PI3K/Akt/NF-ΚB Signaling Pathway. Neurochem. Int..

[62] Salemi M., Vandamme A.M., Guano F., Gradozzi C., Cattaneo E., Casoli C., Bertazzoni U. (1996). Complete Nucleotide Sequence of the Italian Human T-Cell Lymphotropic Virus Type II Isolate Gu and Phylogenetic Identification of a Possible Origin of South European Epidemics. J. Gen. Virol..

[63] Babicki S., Arndt D., Marcu A., Liang Y., Grant J.R., Maciejewski A., Wishart D.S. (2016). Heatmapper: Web-Enabled Heat Mapping for All. Nucleic Acids Res..

[64] Aoki-Kinoshita K.F., Kanehisa M. (2007). Gene Annotation and Pathway Mapping in KEGG. Methods Mol. Biol..

[65] Schindelin J., Arganda-Carreras I., Frise E., Kaynig V., Longair M., Pietzsch T., Preibisch S., Rueden C., Saalfeld S., Schmid B. (2012). Fiji: An Open-Source Platform for Biological-Image Analysis. Nat. Methods.

[66] Turci M., Lodewick J., Di Gennaro G., Rinaldi A.S., Marin O., Diani E., Sampaio C., Bex F., Bertazzoni U., Romanelli M.G. (2012). Ubiquitination and Sumoylation of the HTLV-2 Tax-2B Protein Regulate Its NF-ΚB Activity: A Comparative Study with the HTLV-1 Tax-1 Protein. Retrovirology.

[67] Fochi S., Orlandi E., Ceccuzzi L., Rodolfo M., Vergani E., Turco A., Romanelli M.G., Gomez-Lira M. (2021). Identification of Suitable MRNAs and MicroRNAs as Reference Genes for Expression Analyses in Skin Cells under Sex Hormone Exposure. Gene.

[68] Livak K.J., Schmittgen T.D. (2001). Analysis of Relative Gene Expression Data Using Real-Time Quantitative PCR and the 2(-Delta Delta C(T)) Method. Methods.

