# Supplementary material for: A Panel of Eight miRNAs Is Deregulated in HTLV-2 Infected PBMCs and BJABGu Cell Line
Source: Int J Mol Sci. 2022 Jul 8;23(14):7583. doi: 10.3390/ijms23147583 (PMC9320395; doi:10.3390/ijms23147583)
Supplement: Supplementary file 1 [file ijms-23-07583-s001.zip › ijms-1782899-supplementary.pdf]

International Journal of *Molecular Sciences*

MDPI

Article

A Panel of Eight miRNAs is Deregulated in HTLV-2 Infected PBMCs and BJABGu Cell Line

Elisabetta Pilotti[1], Attilio Cannata[2], Giacomo Magnani[3], Fabio Bignami[4], Andrea Corsi[1], Maria Teresa Valenti[1], Mariam Shallak[5], Greta Forlani[5] and Maria Grazia Romanelli [1,*]

1 Department of Neurosciences, Biomedicine and Movement Sciences, University of Verona, 37124 Verona, Italy; elisabetta.pilotti@univr.it; andrea.corsi@univr.it; mariateresa.valenti@univr.it; mariagrazia.romanelli@univr.it

2 L.C. Laboratori Campisi srl, Avola, SR, Italy; attilio.cannata@gmail.com

3 Unit of Infectious Diseases, Azienda USL-IRCCS, Reggio Emilia, Italy; giacomo.magnani51@gmail.com

4 Department of Clinical Sciences, University of Milano, Milano, Italy; fabio.bignami@gmail.com

5 Laboratory of General Pathology and Immunology "Giovanna Tosi", Department of Medicine and Surgery, University of Insubria, 21100 Varese, Italy; mshallak@uninsubria.it; greta.forlani@uninsubria.it

* Correspondence: mariagrazia.romanelli@univr.it

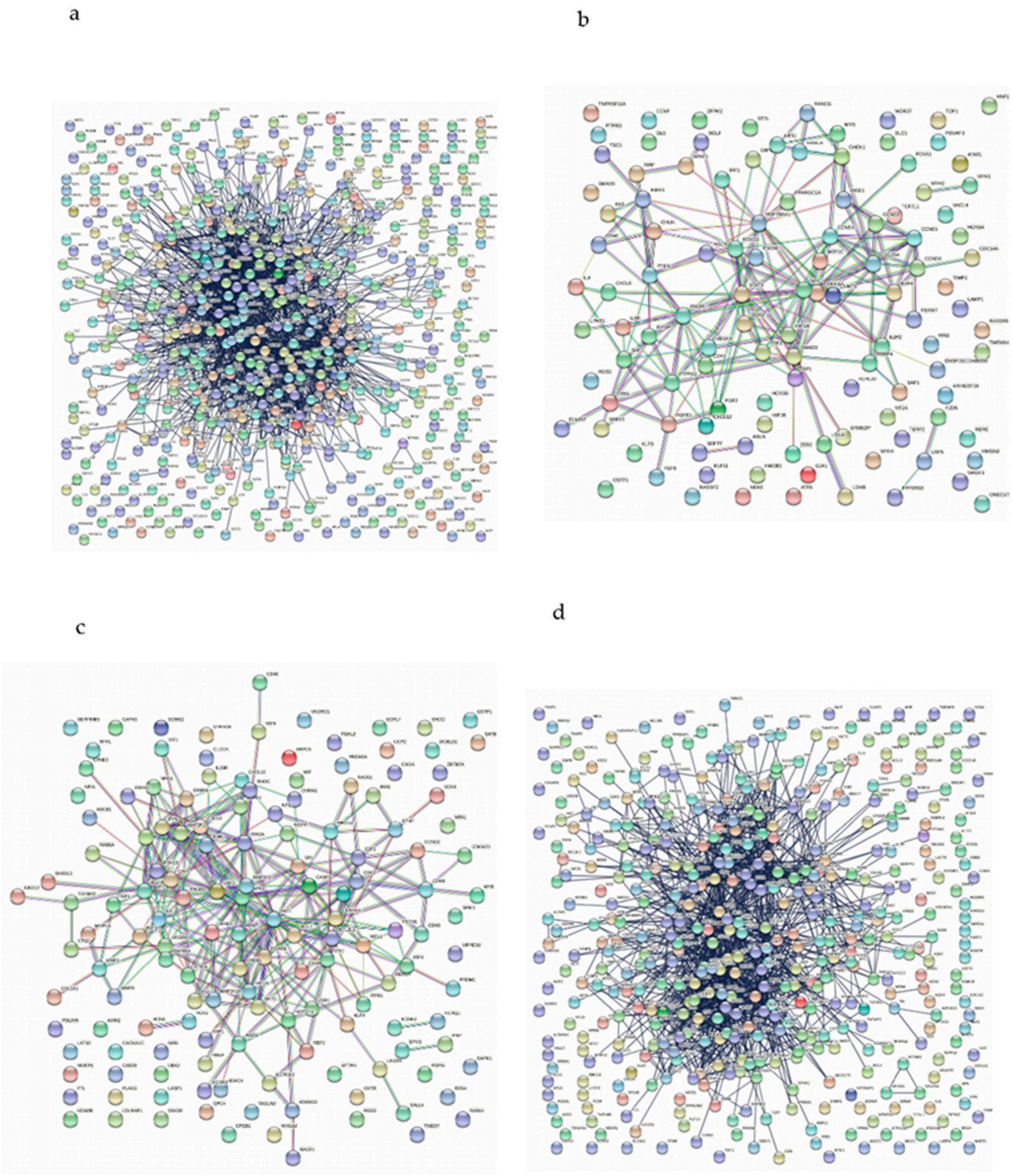

Figure S1. PPIs networks of target genes of 226 differentially expressed miRNAs displayed as follows: a) upregulated miRNAs in HTV-2-infected PBMCs; b) downregulated miRNAs in HTLV-2-infected PBMCs; c) upregulated miRNAs in BJABGu cells; d) downregulated miRNAs in BJABGu cells. Overview of the number of both nodes and edges, average node degree, average local clustering coefficient, and PPI enrichment p-value.

Table S1. STRING networks of target genes of 226 differentially expressed miRNAs.

		N° nodes	N° edges	Average node degree	Average local clustering coefficient	PPI enrichment p-value
Gene set modulated by miRNAs	upregulated in infected PBMCs	584	2043	7	0.387	<1.0e-16
	downregulated in infected PBMCs	114	197	3.46	0.355	<1.0e-16
	upregulated in BJABGu	147	285	3.88	0.339	<1.0e-16
	downregulated in BJABGu	429	1334	6.22	0.365	<1.0e-16
